# Accuracy Evaluation of a Three-Dimensional Face Reconstruction Model Based on the Hifi3D Face Model and Clinical Two-Dimensional Images

**DOI:** 10.3390/bioengineering11121174

**Published:** 2024-11-21

**Authors:** Yujia Xiao, Bochun Mao, Jianglong Nie, Jiayi Liu, Shuo Wang, Dawei Liu, Yanheng Zhou

**Affiliations:** 1 Department of Orthodontics, Peking University School and Hospital of Stomatology, National Center for Stomatology, National Clinical Research Center for Oral Diseases, National Engineering Research Center of Oral Biomaterials and Digital Medical Devices, Beijing Key Laboratory of Digital Stomatology, Research Center of Engineering and Technology for Computerized Dentistry Ministry of Health, Beijing 100081, China; 1710303128@pku.edu.cn (Y.X.); heyyoobc@bjmu.edu.cn (B.M.); 1810303110@pku.edu.cn (J.L.); wangshuo_0724@sina.com (S.W.); liudawei@bjmu.edu.cn (D.L.); 2 School of Software and Microelectronics, Peking University, Beijing 100091, China; jianglongnie@stu.pku.edu.cn

**Keywords:** face, orthodontics, imaging, three dimensional, image reconstruction

## Abstract

Three-dimensional (3D) facial models have been increasingly applied in orthodontics, orthognathic surgery, and various medical fields. This study proposed an approach to reconstructing 3D facial models from standard orthodontic frontal and lateral images, providing an efficient way to expand 3D databases. A total of 23 participants (average age 20.70 ± 5.36 years) were enrolled. Based on the Hifi3D face model, 3D reconstructions were generated and compared with corresponding face scans to evaluate their accuracy. Root mean square error (RMSE) values were calculated for the entire face, nine specific facial regions, and eight anatomical landmarks. Clinical feasibility was further assessed by comparing six angular and thirteen linear measurements between the reconstructed and scanned models. The RMSE of the reconstruction model was 2.00 ± 0.38 mm (95% CI: 1.84–2.17 mm). High accuracy was achieved for the forehead, nose, upper lip, paranasal region, and right cheek (mean RMSE < 2 mm). The forehead area showed the smallest deviation, at 1.52 ± 0.88 mm (95% CI: 1.14–1.90 mm). In contrast, the lower lip, chin, and left cheek exhibited average RMSEs exceeding 2 mm. The mean deviation across landmarks was below 2 mm, with the Prn displaying the smallest error at 1.18 ± 1.10 mm (95% CI: 0.71–1.65 mm). The largest discrepancies were observed along the Z-axis (Z > Y > X). Significant differences (*p* < 0.05) emerged between groups in the nasolabial, nasal, and nasofrontal angles, while the other 13 linear and 3 angular measurements showed no statistical differences (*p* > 0.05). This study explored the feasibility of reconstructing accurate 3D models from 2D photos. Compared to facial scan models, the Hifi3D face model demonstrated a 2 mm deviation, with potential for enriching 3D databases for subjective evaluations, patient education, and communication. However, caution is advised when applying this model to clinical measurements, especially angle assessments.

## 1. Introduction

With the development of orthodontics, there is a growing emphasis among orthodontists and patients on not only teeth alignment and occlusion but also on facial soft tissue changes [1]. To accurately evaluate the changes in soft tissues during orthodontic treatment, an increasing number of orthodontists are using models obtained from 3D facial scans for research purposes [2,3]. However, a substantial collection of two-dimensional (2D) images has been amassed in orthodontic clinical practice. If these 2D images could be converted into clinically available 3D models, the clinical applicability of 3D facial analysis would be greatly enhanced.

In today’s computer vision (CV) research field, 3D face reconstruction is a popular topic. Its applications span the gaming and film industries, security authentication, and face recognition, among others [4]. Due to the complex details within 3D facial structures, their reconstruction process presents significant challenges and is susceptible to variations in illumination, expression, and posture. Improving model robustness is therefore a priority in reconstruction using CV methods [5]. Orthodontic facial photography, conducted under strict and standardized conditions, supports the enhancement of robustness [6].

The three-dimensional morphable model (3DMM) is a classic algorithm used for 3D face reconstruction, first proposed by Blanz and Vetter [7] in 1999. Its core principle involves creating a 3D facial model through shape and texture parameters, thereby generating a model resembling a real human face by solving for the optimal linear combinations of these parameters. Recent developments in deep learning have led to its integration with 3D facial modelling techniques, such as the Hifi3Dface model [8]. This method introduces an innovative geometric template, generating highly realistic 3D facial structures through detail synthesis, regional pyramid bases, and localized fitting. Compared to traditional 3DMM, this approach better captures facial geometry with only linear bases, achieving a more lifelike reconstruction.

In addition, other 3D face reconstruction algorithms, such as epipolar geometry (EG), shape-from-shading, and one-shot learning (OSL), have seen significant development in recent years. Anbarjafar et al. [9] segmented the face into four regions using 68 facial landmarks, achieving strong generalization. Jiang et al. [10] employed a shape-from-shading technique inspired by face animation, incorporating RGB-D and monocular video data. Xing et al. [11] applied a one-shot learning reconstruction method to create 3D facial models from a single image.

High-precision 3D facial scanners are commonly used in orthodontic studies, particularly for measuring facial symmetry and monitoring soft tissue changes in patients [12,13]. Additionally, facial scanning is extensively applied in the field of digital prosthetics, enabling users to capture complex movements for precise jaw motion analyses [14]. These scanners employ three primary imaging technologies: lasers, structured light, and stereophotography [15]. Research indicates that the root mean square error (RMSE) for these 3D scanners is approximately 0.5 mm, even reaching as low as 0.05 mm for certain brands [16]. Specifically, the FaceSCAN 3D scanner, which is primarily reliant on structured light technology, yields a scanning error of approximately 0.2 mm, as noted in previous studies [16,17]. However, its substantial size and high cost restrict its application. In fact, the purpose of our study is not to replace 3D facial scanners. Instead, we aim to use this method to convert scarce and valuable historical 2D data into 3D models, thereby enriching the orthodontic research database. These models can be used for subjective evaluations, patient education, communication, and so on.

In the present study, we explore the automatic generation of 3D face models using two 2D facial photos, basing their generation on the Hifi3Dface method preliminarily. Subsequently, we compare this reconstruction model to the FaceSCAN model and measure the 3D deviation in the results to assess the accuracy of this modelling. In addition, we measure 19 soft tissue items to assess their clinical feasibility.

The null hypothesis was that there were no statistically significant differences between the face scan models and 3D reconstruction model in clinical accuracy and feasibility.

## 2. Materials and Methods

### 2.1. Sample Selection

This study was approved by the Institutional Review Board of Peking University School and Hospital of Stomatology (PKUSSIRB-202058135). The involved patients were treated at Peking University Stomatology Hospital (China) from January to April 2021. According to Jacob Cohen’s statistical book [18], we selected a high effect size of 0.8 for Cohen’s d. With a test power of 0.95 and a significance level of α = 0.05, the calculated minimum sample size for this paired design study was 23 participants. The study ultimately included a total of 23 participants, consisting of 14 females and 9 males, with an average age of 20.70 ± 5.36 years. All participants supplied their written consent.

The criteria for inclusion include (1) Han Chinese individuals, aged between 12 and 35 years, with a complete set of permanent teeth and (2) complete medical information, including 2D photographs, 3D models, etc.

The criteria for exclusion include (1) previous orthognathic surgery; (2) maxillofacial tumours; (3) cleft lip and cleft palate or other developmental anomalies; (4) neurological disorders (e.g., facial paralysis); (5) facial scarring; and (6) significant facial asymmetry.

### 2.2. Acquisition of 2D Photographs and 3D Scans

The process of collecting 2D photos and 3D data was based on that of Mao et al. [19]. The FaceSCAN3D System (3DShape, Erlangen, Germany) was used to capture the 3D models. To acquire 2D photos, a Canon camera (EOS 60D, 60 mm fixed-focus lens) was used, set to a shutter speed of 1/125, aperture of F7.1, and ISO of 100. Both 2D photos and 3D facial scans were obtained on the same day.

### 2.3. Three-Dimensional Model Reconstruction

Three-dimensional face reconstruction was performed with frontal and lateral images using the open-source Hifi3D face model. This detailed process involved several key steps.

#### 2.3.1. Initial Model Fitting

We used a linear 3DMM based on Principal Component Analysis (PCA) as our initial model. The shape and albedo variables were expressed as

(1) $$s=\bar{s}+Sx_{\mathrm{shp}}$$

(2) $$a=\bar{a}+Ax_{\mathrm{alb}}$$

where $\bar{s}$ is the average 3D face shape, in vector format; *S* is the basis for shape recognition; $\bar{a}$ is the mean reflectivity graph, in vector form; *A* is the albedo graph’s basis; and x*_shp_* and x*_alb_* are the corresponding parameter vectors to be estimated. The detected 3D landmarks (including depth information) were fitted to the initial shape model using the ridge regression method. Some texture maps were extracted by projecting the shape model onto each input image. Then, the partial texture maps were mixed into full texture maps using a Laplacian pyramid technique. The initial albedo parameter was fitted to the mixed texture based on another ridge regression step.

Next, we fitted the initial shape model to the detected 3D landmarks. A partial texture map was produced by projecting the shape model onto each input image.

#### 2.3.2. Optimization

The optimization parameters were defined as follows:

P = {x*_shp_*, x*_alb_*, x*_light_*, x*_pose_*} (3)

In this formula, x*_shp_* is the shape parameter, x*_alb_* is the reflectance parameter, x*_light_* is the second-order spherical harmonic illumination parameter, and x*_pose_* includes the rotation and translation parameters for rigid transformation. For each view, 3DMM parameters x*_shp_* and x*_alb_* for the user and the lighting parameters x*_light_* and x*_pose_* were estimated. The constraints include landmark loss L*_lan_*, RGB photoloss L*_rgb_*, depth loss L*_dep_*, and perceptual identity loss L*_id_*.

#### 2.3.3. Morphable Model Augmentation

The morphable model augmentation (MMA) method works as follows. First, data generation and perturbation are performed. A baseline 3DMM was chosen. The baseline model was then perturbed and deformed to generate additional facial models. This involved replacing the nose and mouth regions with models from other sources, applying rotation perturbations, and using rigid transformations such as rotation, translation, and scaling. Additionally, the generated facial models were mirrored. These steps resulted in the generation of many facial models.

The iterative 3DMM construction algorithm consists of two levels of iteration. In the outer loop, a subset of models is randomly selected from the generated dataset as the test set. In the inner loop, the current 3DMM is used to fit the models in the test set, and the models with the highest fitting errors are selected and added to the model collection. The basis vectors of the 3DMM are then recomputed based on the models in the collection. The inner loop is iterative, continuously improving the expressiveness of the 3DMM. The goal of this algorithm is to capture as many data variations as possible with few principal components.

MMA enhanced the capacity and expressiveness of the 3DMM. By introducing diverse data samples and perturbation operations, MMA could better capture the asymmetry and variations in facial shapes. Furthermore, the iterative construction algorithm continually improved the expressiveness of the 3DMM by optimizing the results of the PCA.

#### 2.3.4. Face Reflex Synthesis

To enhance facial detail, we tested a hybrid approach that integrated high-resolution synthetic maps with normal maps. Traditional super-resolution-based methods can effectively capture features like eyebrows and hair, yet directly generating high-resolution texture maps can sometimes yield overly detailed, unrealistic results. By using pyramid parameters, this method achieved a balance between realism and detail.

First, we divided facial regions into 8 subregions, indicated by the different colours in the UV map. Region partitioning was performed because different regions are characterized by different types of skin/hair details. Second, with the regional pyramid bases, the different types of skin/hair details in different regions were separately preserved via high-resolution bases, and the low-resolution fitting process allowed the algorithm to focus on major facial structures and the shapes of the eyebrows and lips. Finally, two refinement networks were applied to synthesize the details from the albedo and normal maps.

### 2.4. Three-Dimensional Deviation Measurement

The alignment of the 3D reconstructed models and the face scans was performed using Cliniface software (v5.2.1, Unlocking Facial Clubs, Perth, Australia). A 3D coordinate system was established with the midpoint of the bilateral tragion as the origin, while the median sagittal plane was defined by all midpoints of the face, and the Frankfurter horizontal plane was taken as the horizontal plane. The X-, Y-, and Z-axes corresponded to the left, superior, and anterior directions, respectively.

**Figure 1:**
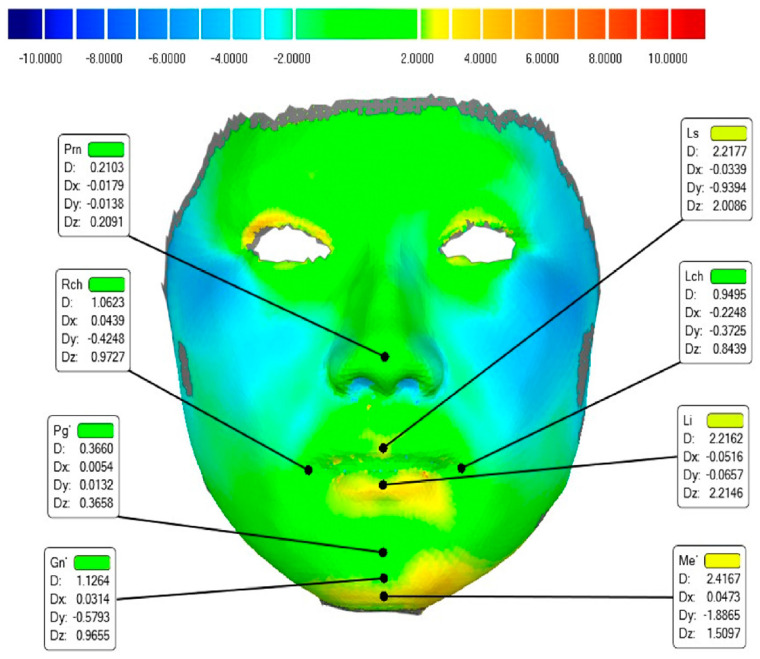
Colour-coded deviation analysis map of a typical subject. The deviation of all the landmarks is shown as follows: Prn = pronasale; Ls = labrale superius; Li = labrale inferius; Lch = left cheilion; Rch = right cheilion; Pg’ = pogonion of soft tissue; Gn’ = gnathion of soft tissue; and Me’ = menton of soft tissue.

After repositioning, model registration and deviation analysis were conducted through Geomagic Studio 2013 software (Geomagic, Morrisville, North Carolina, USA). The alignment registration method followed that of Mao et al. [19]. First, the bilateral canthus, pronasale, and soft tissue nasion were manually marked for pre-alignment. Then, a best-fit alignment was performed. Additionally, the facial area was divided into nine regions according to Wang [20]: the forehead, left and right para-nasal areas, left and right cheeks, nose, upper lip, labium, and chin. To assess the deviations across the face and nine areas, colour mapping and an RMSE analysis were performed. Finally, the 3D accuracy for Prn, Ls, Li, Lch, Rch, Pg’, Gn’, and Me’ was calculated (Figure 1). In order to assess the consistency of the landmarks, we randomly selected 7 of the 23 patients to repeatedly measure the 3D deviation of these 8 landmarks and calculate their interclass correlation coefficient (ICC) (Table 1).

### 2.5. Soft Tissue Measurements

**Figure 2:**
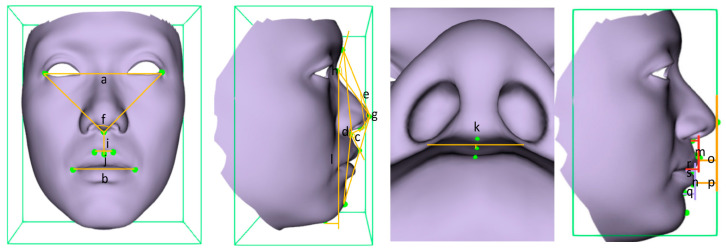
Three-dimensional soft tissue measurements. (a) Outercanthal Width; (b) Labial Fissure Width (ChiL-ChiR); (c) nasolabial angle (Prn-Sn-Ls); (d) Facial Convexity (Gl-Sn-Pg’); (e) Total Facial Convexity (Gl-Prn-Pg’); (f) Outer Canthal, Nasal Angle; (g) Nasal Angle (N’-Prn-Sn); (h) Nasofrontal Angle (Gl-N’-Prn); (i) Philtral Length (Sn-Ls); (j) Philtral Width (CphR-CphL); (k) Philtral Depth; (l) Facial Height (N’-Gn’); (m) Upper Lip Height (Sn-Stos); (n) Lower Lip Height (Stoi-Sl); (o) Upper lip Protrusion (|Prn-Ls|z); (p) Lower lip Protrusion (|Prn-Li|z); (q) Mentolabial furrows depth (|Li-Sl|z); (r) Thickness of upper vermilion (|Ls-Stos|z); (s) Thickness of lower vermilion (|Li-Stoi|z).

The “Measurements Browser” and “Add Calliper Measurements” tools in Cliniface 5.2.1 were used for soft tissue measurements (Table 2, Figure 2). All data were collected by two orthodontists, with repeated measurements taken one week apart and the average of the results computed.

### 2.6. Statistical Analysis

All the data were analyzed using SPSS (v26.0). The Shapiro–Wilk test was applied to verify normality for all soft tissue measurements, confirming their normal distribution (*p* > 0.05). Intragroup and intergroup consistency tests were performed on these measurements with 2-way absolute agreement, followed by paired *t* tests to ascertain the differences between the two sets of models. The significance level was set at α = 0.05, and differences were considered statistically significant when *p* < 0.05.

## 3. Results

### 3.1. Three-Dimensional Deviation Analysis Results for the Full Face and Nine Regions

**Figure 3:**
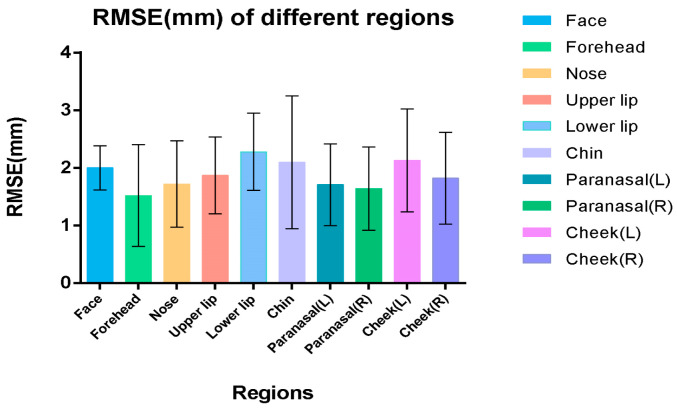
Root mean square error (RMSE) of different regions of the reconstruction models.

**Figure 4:**
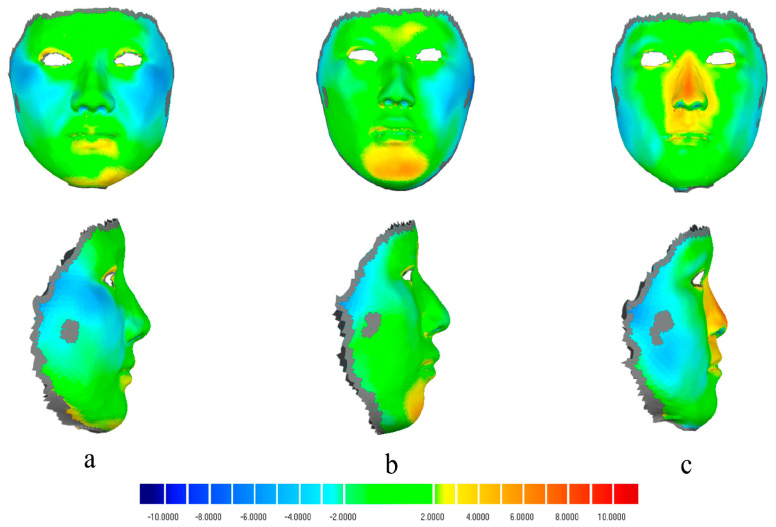
Cloud maps depicting 3D deviations between face scan models and reconstructions for three patients with varied facial profiles: straight (**a**), concave (**b**), and convex (**c**).

The root mean square error (RMSE) for the Hifi3Dface-based reconstruction model averaged 2.00 ± 0.38 mm (95% CI: 1.84–2.17 mm) (Table 3, Figure 3). High reconstruction accuracy was observed for the forehead, nose, upper lip, paranasal region, and right cheek, with an average RMSE of less than 2 mm. The labium, chin, and left cheek exhibited average RMSEs greater than 2 mm, which may be related to the significant differences in the lower facial region across different sagittal skeletal types. Additionally, the zygomatic area appeared relatively retruded compared to the scan model, with the brow arch being more pronounced externally. In patients with convex profiles, the chin appeared more pronounced, while in those with concave profiles, nasal features were more prominent. (Figure 4)

**Figure 5:**
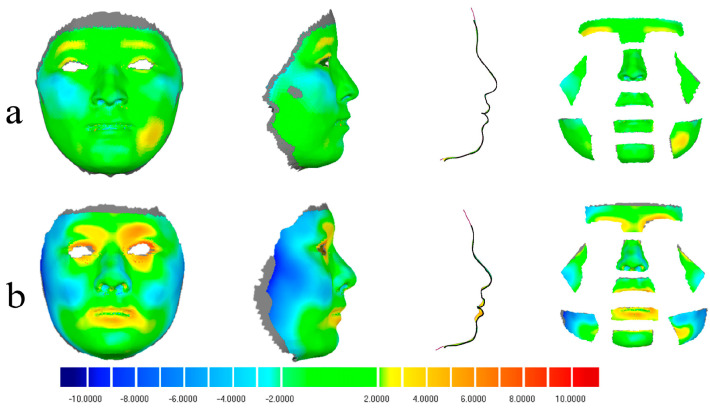
Best and worst reconstruction results: (**a**) best reconstruction outcome; (**b**) worst reconstruction outcome.

Figure 5 illustrates both the best and worst reconstruction outcomes, with the RMSE for the best reconstruction measuring 1.36 mm across the face and under 2 mm in the nine distinct regions. Notably, the RMSE around the cheek reached 0.81 mm. In the worst case, the RMSE was 2.73 mm around the entire face, reaching 3 mm for the labium and cheek.

### 3.2. Three-Dimensional Deviation of Landmarks

**Figure 6:**
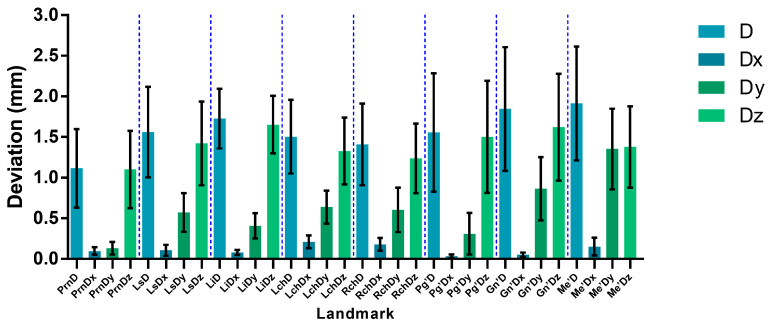
Three-Dimensional deviation of eight landmarks (mean ± 95% CI, mm; D: deviation; Dx: horizontal deviation; Dy: vertical deviation; Dz: sagittal deviation; Prn: pronasale; Ls: labrale superius; Li: labrale inferius; Lch: left cheilion; Rch: right cheilion; Pg’: pogonion of soft tissue; Gn’: gnathion of soft tissue; Me’: menton of soft tissue).

As shown in Table 1, all landmarks have an ICC above 0.8, indicating good consistency in the results. The 3D deviations for each landmark in the reconstruction models are displayed in Figure 6 and Table 4 and compared to the face scans, with errors of <2 mm, which is within the clinically acceptable limits. Landmarks such as Prn, LCh, and RCh demonstrated the highest accuracy, while Gn’ and Me’ showed slightly larger deviations but still stayed below 3 mm. Deviations along the Y-axis, excluding Me’, were generally under 1 mm. The largest discrepancies appeared in the Z-axis direction (Z > Y > X) and largely contributed to landmark deviations.

### 3.3. Results of Soft Tissue Measurements

The ICC results are presented in Table 5, showing that the intragroup and intergroup average ICC values exceed 0.90 for all the measured features, indicating the high reliability of the measurements. Table 6 shows the outcomes of these measurements. Sixteen out of nineteen soft tissue areas displayed no statistically significant differences (*p* > 0.05), especially among all linear measurements. However, significant differences were observed in angular measurements, notably for the nasolabial angle (*p* < 0.05). Among all characteristics, the average errors in the linear measurements remained below 1 mm, while those in angular measurements were high as 4.81°.

## 4. Discussion

In the present study, the original code of the Hifi3D face was modified to include both front and lateral facial images, and 3D facial models were successfully reconstructed. To assess the error between the reconstructed models and the FaceSCAN model, we adopted a 2 mm criterion as the minimum standard for evaluation. High accuracy was achieved across all regions except for the lower lip, chin, and left cheek, with a mean RMSE < 2 mm. As for the soft tissue measurements, 13 linear and 3 angular measurements showed no significant differences (*p* > 0.05), except for in the nasolabial, nasal, and nasofrontal angles. The null hypothesis could not be fully confirmed. Currently, there is no uniform standard for the clinical deviation of soft tissue. Deviations ≥ 3 mm should be considered clinically relevant [21], with 1–3 mm deviations deemed relevant only in extremely detailed evaluations for micro-esthetic purposes. In addition, different studies [22,23] have suggested that for facial soft tissues, an error lower than 2 mm is considered clinically acceptable. In addition, Mai et al. [24] found that some handheld 3D facial scanners exhibit a size deviation of approximately 1.5 mm.

To minimize the impact of software operations on measurements, we used Cliniface software for 3D model relocation, landmark positioning assistance, and soft tissue measurements. Cliniface is based on the open-source MATLAB toolbox, MeshMonk [25], which introduces a certain degree of error. An error of 0.2 mm for multiple registrations of the template onto the same facial images has been reported [26]. Nevertheless, several studies [27,28,29] have demonstrated that automatic landmark detection methods can achieve precision comparable to, or even exceeding, that of manual landmark positioning. White et al. [30] found that the mean inter-observer error in manual landmarking was 0.40 mm, while the variation in automatic landmark indication averaged 0.27 mm. The operator’s clinical orthodontic experience and software proficiency also affect the accuracy of landmark positioning. To address this, operators received relevant software training before conducting the measurements. Furthermore, our study involves an inherent degree of unassessable selection bias. Even so, based on the current ICC results, our error remains within an acceptable range.

As the results show, we established 19 soft tissue indicators. Typically, linear distance variations should be within the clinically acceptable range of 1 mm (*p* > 0.05). However, regarding angular differences, three metrics exhibited statistically significant disparities, with variations reaching up to 4.81°. The differences in the nasolabial and nasal angles can be attributed to the higher and more prominent nose and fuller upper lip in the Caucasian template than in the Asian template. The clinical application of these models requires further consideration.

The primary landmark deviation was 2.00 ± 0.38 mm, mainly concentrated in the Z-axis (sagittal) direction, suggesting room for further depth accuracy improvements. Currently, RGB-D cameras can simultaneously capture depth maps and corresponding colour images during photo capture [31]. Pixel value data, through coordinate transformations, can be transformed into point cloud data, yielding a 3D reconstruction model. This is similar to the basic principle used for some 3D scanners. However, regular cameras cannot capture depth information in their images. Therefore, one fundamental task in 3D facial reconstruction is to compensate for the loss of depth information.

In the field of CV, many studies have attempted to perform 3D reconstructions from single images, but they have mainly focused on morphology, expressions, and facial skin textures. Few articles have addressed the accuracy of soft tissue modelling in the middle and lower face regions [32]. Moreover, limited by the sample size and quality of 3D datasets, the extrapolatability of the code has emerged as the greatest problem for 3D reconstruction. Recent studies have used multiple images for 3D face reconstruction [33,34], typically employing 45° side images, as most landmarks can be aligned across different perspectives. Lium et al. [35] utilized a CNN for 3D facial reconstruction with frontal and lateral images, achieving a mean normalized mean error NME of 0.0164, but an evaluation of the perioral region was not conducted. Garrido et al. [36] focused on lip reconstruction, with an average error of 3 mm reported. Additionally, prior studies indicated that facial expressions, such as mouth opening, may lead to 5–10 mm deviations in the lip area [37].

In forensic medicine [38], many researchers have previously reconstructed 3D soft tissue models with CBCT data, but less accuracy than in our study was observed. Laura et al. [39] used CBCT data to conduct 3D reconstructions, yielding errors within 2.5 mm for 70.9% of their meshes. However, the error rose to 3.7–5.5 mm around the mouth and 3–7 mm near the nose. Additionally, their landmark errors ranged from 0.2 mm to 2.4 mm. Qiu et al. [40] reported an improvement in their reconstruction results using CBCT data, with a facial RMSE of approximately 1.5 mm, but the extrapolation performance of the approach was poor, with an error of 2.68 mm.

High-precision 3D facial remodelling has been achieved [19], using manual modelling software, that surpasses our method, albeit with greater complexity and a reliance on practitioner expertise. In contrast, our proposed method allows for the automatic generation of 3D face models in a short timeframe by inputting frontal and lateral images, thus eliminating the need for meticulous adjustments based on experience.

During the 3D reconstruction process, we utilized the original facial template from the Hifi3Dface model, which was based on data from Caucasian individuals [8]. When applied to Asian individuals, discrepancies in the facial features emerged, leading to the final results retaining some characteristics typical of Caucasian features. In general, the brow bone and chin were relatively prominent in the 3D reconstructed models, while the cheek was more recessed. In addition, the plumpness of soft tissue in the cheek region posed challenges in matching depth information, leading to poor reconstruction in this area. Moreover, in individuals with different sagittal skeletal types, the morphology of their lower facial profile varies significantly, with notable differences in chin curvature. For patients with a skeletal Class II malocclusion, the mandible is retrusive, whereas skeletal Class III cases exhibit mandibular prognathism. In addition, the limited number of chin landmarks further increased the difficulty of reconstructing the lower lip and chin. During the initial model fitting process of the Hifi3dFace model, alignment was initially conducted from top to bottom. As a result, deviations were relatively minimal in the forehead area, but there were more pronounced deviations in the chin and lower lip regions.

The lack of 45° lateral photos and images from other angles increased the complexity of the 3D reconstruction model. Notably, in general, as the number of acquired photos increases, the representation of depth information becomes more easily refined. For this reason, in the field of CV today, screenshots from videos are frequently used for reconstructing 3D facial images [41]. In orthodontic clinical practice, 90° lateral images are typically used, but only half of the landmarks seen in lateral images can be used for fitting. This limitation highlights a direction for future algorithmic advancements in computer vision.

Moreover, this study has other limitations. Patients with significant facial asymmetry or scars were not included, which limits the model’s applicability. Additionally, this study is a preliminary attempt with a small sample size of only 23 Chinese participants, so the findings may primarily apply to Han Chinese individuals. Since generalizing to other ethnicities may introduce bias, larger sample sizes will be necessary for more detailed assessments in future clinical research. The purpose of this study was to automate the conversion of images to 3D models and to preliminarily explore the clinical practicality of reconstruction models. Based on the results, higher-precision models should be explored in the future by integrating orthodontic clinical knowledge and advanced algorithms into computer vision. Future studies could focus on developing facial templates tailored to different ethnic groups to optimize 3D face reconstruction across diverse populations, enhance the code’s generalizability, and broaden its potential clinical applications.

## 5. Conclusions

This study integrated a CV method into the medical field for efficient 3D model reconstruction, and the obtained modelling results displayed a difference of approximately 2 mm compared to face scans. Nevertheless, caution is advised when applying the proposed method in clinical orthodontics. Further research is necessary to improve the accuracy of the reconstruction models obtained.

## Figures and Tables

**Table 1:** Consistency of the eight landmarks (ICC: intraclass correlation coefficient; Prn: pronasale; Ls: labrale superius; Li: labrale inferius; Lch: left cheilion; Rch: right cheilion; Pg’: pogonion of soft tissue; Gn’: gnathion of soft tissue; Me’: menton of soft tissue).

ICC	Prn	Ls	Li	Rch	Lch	Pg’	Gn’	Me’
total	0.968	0.978	0.873	0.898	0.981	0.968	0.973	0.967
x	0.871	0.866	0.902	0.838	0.870	0.944	0.872	0.959
y	0.806	0.953	0.889	0.869	0.924	0.882	0.958	0.944
z	0.965	0.969	0.818	0.887	0.952	0.967	0.970	0.942

**Table 2:** Items for 3D soft tissue measurements ((L, R) Chi = (Left, Right) Cheilion; Sn = Subnasal; Stos = Stomion superius; Stoi = Stomion inferius; Sl = Sublabial; (L, R) Cph = (Left, Right) Crista Philtri; Gl = Glabella; N’ = Nasion of soft tissue).

Measurement Index	Definition
Outercanthal Width	Horizontal distance between the lateral canthi
Labial Fissure Width (ChiL-ChiR)	Distance between the mouth commissures
Nasolabial Angle (Prn-Sn-Ls)	Angle at Sn subtended by side Prn–Ls
Facial Convexity (Gl-Sn-Pg’)	Angle at Sn subtended by side Gl–Pg’
Total Facial Convexity (Gl-Prn-Pg’)	Angle at Prn subtended by side Gl–Pg’
Outer Canthal, Nasal Angle	Angle at Sn subtended by the outer canthi
Nasal Angle (N’-Prn-Sn)	Angle at Prn subtended by side N’–Sn
Nasofrontal Angle (Gl-N’-Prn)	Angle at N’ subtended by side Gl–Prn
Philtral Length (Sn-Ls)	Distance from nasal bone/base to the midline of the upper lip vermilion border
Philtral Width (CphR-CphL)	Distance between philtral ridges, measured just above the vermilion border
Philtral Depth	Depth measured at the deepest midline point between philtral ridges
Facial Height (N’-Gn’)	Vertical height (length) of the face (N’-Gn)
Upper Lip Height (Sn-Stos)	Vertical distance between Sn and Stos
Lower Lip Height (Stoi-Sl)	Vertical distance between Stoi and Sl
Upper Lip Protrusion (|Prn-Ls|z)	Sagittal distance between Prn and Ls
Lower Lip Protrusion (|Prn-Li|z)	Sagittal distance between Prn and Li
Mentolabial Furrow Depth (|Li-Sl|z)	Sagittal distance between Li and Sl
Thickness of Upper Vermilion (|Ls-Stos|z)	Sagittal distance between Ls and Stos
Thickness of Lower Vermilion (|Li-Stoi|z)	Sagittal distance between Li and Stoi

**Table 3:** The deviations in each region between the face scan models and 3D reconstruction models (mm) (SD: standard deviation; CI: confidence interval).

Area	Face	Forehead	Nose	Upper Lip	Lower Lip	Chin	Paranasal (L)	Paranasal (R)	Cheek (L)
Mean	2.00	1.52	1.72	1.87	2.28	2.10	1.71	1.64	2.13
SD	0.38	0.88	0.75	0.67	0.67	1.15	0.71	0.72	0.89
Lower 95%CI	1.84	1.14	1.39	1.58	1.99	1.60	1.40	1.33	1.74
Upper 95%CI	2.17	1.90	2.04	2.16	2.57	2.59	2.01	1.95	2.52

**Table 4:** Deviations of landmarks in different directions (SD: standard deviation; CI: confidence interval; D: total deviation; Dx: deviation in horizontal level; Dy: deviation in vertical level; Dz: deviation in sagittal level).

	D				Dx				Dy				Dz			
	Mean	SD	95%CI		Mean	SD	95%CI		Mean	SD	95%CI		Mean	SD	95%CI	
Prn	1.18	1.10	0.71	1.65	0.10	0.11	0.06	0.15	0.14	0.18	0.06	0.21	1.16	1.08	0.70	1.63
Ls	1.61	1.25	1.08	2.15	0.11	0.15	0.04	0.17	0.57	0.55	0.33	0.81	1.48	1.15	0.98	1.97
Li	1.78	0.77	1.44	2.11	0.08	0.07	0.05	0.11	0.43	0.35	0.28	0.58	1.70	0.75	1.37	2.02
Lch	1.53	1.02	1.10	1.97	0.21	0.18	0.14	0.29	0.65	0.46	0.45	0.85	1.35	0.92	0.95	1.75
Rch	1.49	1.13	1.01	1.98	0.18	0.18	0.11	0.26	0.63	0.62	0.37	0.90	1.32	0.96	0.90	1.73
Pg’	1.58	1.66	0.86	2.30	0.04	0.05	0.02	0.06	0.31	0.59	0.06	0.57	1.53	1.58	0.84	2.21
Gn’	1.88	1.73	1.13	2.63	0.06	0.06	0.03	0.08	0.89	0.89	0.50	1.27	1.65	1.50	1.00	2.30
Me’	1.98	1.57	1.30	2.66	0.15	0.25	0.05	0.26	1.41	1.11	0.93	1.89	1.41	1.13	0.93	1.90

**Table 5:** Consistency test results of soft tissue measurements between face scan models and 3D reconstruction models (ICC: intraclass correlation coefficient; CI: confidence interval).

Measurement	Intrarater 1 ICC (95%CI)	Intrarater 2 ICC (95%CI)	Interrater ICC (95%CI)
Outercanthal width	0.983 (0.970–0.991)	0.946 (0.904–0.970)	0.960 (0.925–0.978)
Labial fissure width (ChiL-ChiR)	0.934 (0.885–0.981)	0.972 (0.951–0.985)	0.951 (0.908–0.974)
Nasolabial angle (Prn-Sn-Ls)	0.983 (0.969–0.990)	0.990 (0.981–0.994)	0.972 (0.950–0.985)
Facial convexity (Gl-Sn-Pg’)	0.980 (0.960–0.989)	0.991 (0.983–0.995)	0.975 (0.803–0.992)
Total facial convexity (Gl-Prn-Pg’)	0.992 (0.986–0.996)	0.998 (0.997–0.999)	0.966 (0.919–0.984)
Outer canthal, nasal angle	0.967 (0.941–0.981)	0.967 (0.944–0.981)	0.930 (0.867–0.962)
Nasal angle (N’-Prn-Sn)	0.918 (0.856–0.954)	0.990 (0.990–0.995)	0.932 (0.875–0.963)
Nasofrontal angle (Gl-N’-Prn)	0.985 (0.972–0.992)	0.995 (0.990–0.997)	0.984 (0.971–0.991)
Philtral length (Sn-Ls)	0.963 (0.934–0.979)	0.934 (0.882–0.963)	0.941 (0.893–0.967)
Philtral width (CphR-CphL)	0.924 (0.866–0.957)	0.912 (0.818–0.955)	0.957 (0.923–0.976)
Philtral depth	0.945 (0.903–0.969)	0.938 (0.890–0.972)	0.905 (0.829–0.947)
Facial height (N’-Gn’)	0.995 (0.990–0.997)	0.986 (0.975–0.992)	0.962 (0.888–0.983)
Upper lip height (Sn-Stos)	0.935 (0.887–0.964)	0.968 (0.943–0.982)	0.946 (0.902–0.970)
Lower lip height (Stoi-Sl)	0.912 (0.846–0.950)	0.955 (0.918–0.976)	0.942 (0.894–0.968)
Upper lip protrusion (|Prn-Ls|z)	0.970 (0.947–0.983)	0.998 (0.996–0.999)	0.986 (0.975–0.992)
Lower lip protrusion (|Prn-Li|z)	0.926 (0.869–0.958)	0.994 (0.989–0.996)	0.962 (0.832–0.986)
Mentolabial furrows depth (|Li-Sl|z)	0.953 (0.917–0.974)	0.948 (0.908–0.971)	0.939 (0.812–0.974)
Thickness of upper vermilion (|Ls-Stos|z)	0.932 (0.880–0.961)	0.934 (0.884–0.963)	0.919 (0.851–0.956)
Thickness of lower vermilion (|Li-Stoi|z)	0.962 (0.933–0.979)	0.931 (0.878–0.961)	0.951 (0.906–0.974)

**Table 6:** Soft tissue measurements from the face scan models and reconstruction models (*: significance level of *p* < 0.05).

Measurements	Face Scan Model (Mean ± SD)	Reconstruction Model (Mean ± SD)	Deviation of Two Models (Mean ± SD)	*t*	*p*
Outercanthal width	92.95 ± 2.70	92.41 ± 1.26	0.54 ± 2.35	1.102	0.283
Labial fissure width (ChiL-ChiR)	44.77 ± 2.89	45.68 ± 2.80	−0.92 ± 2.79	−1.587	0.129
Nasolabial angle (Prn-Sn-Ls)	105.73 ± 7.61	100.92 ± 5.01	4.81 ± 8.77	2.627	0.015 *
Facial convexity (Gl-Sn-Pg’)	167.16 ± 4.11	167.81 ± 2.47	−0.65 ± 2.86	−1.090	0.288
Total facial convexity (Gl-Prn-Pg’)	144.50 ± 4.42	144.65 ± 2.40	−0.15 ± 3.79	−0.190	0.851
Outer canthal, nasal angle	93.49 ± 2.33	92.69 ± 2.99	0.80 ± 3.27	1.171	0.254
Nasal angle (N’-Prn-Sn)	117.65 ± 3.83	122.31 ± 2.40	−4.66 ± 3.81	−5.865	0.000 *
Nasofrontal angle (Gl-N’-Prn)	142.54 ± 4.18	144.66 ± 2.86	−2.12 ± 4.41	−2.298	0.031 *
Philtral length (Sn-Ls)	14.82 ± 0.93	14.57 ± 1.38	0.24 ± 1.05	1.107	0.280
Philtral width (CphR-CphL)	12.81 ± 0.68	12.42 ± 0.94	0.39 ± 0.91	2.040	0.054
Philtral depth	1.72 ± 0.62	1.53 ± 0.44	0.19 ± 0.71	1.271	0.217
Facial height (N’-Gn’)	115.11 ± 3.68	115.96 ± 2.43	−0.85 ± 3.60	−1.130	0.271
Upper lip height (Sn-Stos)	21.68 ± 1.27	22.18 ± 1.43	−0.50 ± 1.21	−1.960	0.063
Lower lip height (Stoi-Sl)	16.31 ± 1.23	16.43 ± 1.30	−0.12 ± 1.20	−0.464	0.647
Upper lip protrusion (|Prn-Ls|z)	8.33 ± 2.02	7.62 ± 1.63	0.71 ± 2.14	1.587	0.127
Lower lip protrusion (|Prn-Li|z)	11.53 ± 2.59	10.54 ± 1.62	0.99 ± 2.49	1.916	0.068
Mentolabial furrows depth (|Li-Sl|z)	6.54 ± 1.23	6.57 ± 1.10	−0.03 ± 1.14	−0.122	0.904
Thickness of upper vermilion (|Ls-Stos|z)	4.91 ± 1.10	5.00 ± 0.88	−0.09 ± 1.29	−0.321	0.751
Thickness of lower vermilion (|Li-Stoi|z)	2.50 ± 1.18	2.84 ± 1.04	−0.34 ± 1.36	−1.203	0.242

## Data Availability

The datasets analyzed in this study are available from the corresponding author upon reasonable request.
